# Cannabidiol for functional seizures (psychogenic nonepileptic seizures/attacks) and other stress‐associated disorders

**DOI:** 10.1002/epi4.12805

**Published:** 2023-08-18

**Authors:** Ali A. Asadi‐Pooya

**Affiliations:** ^1^ Epilepsy Research Center Shiraz University of Medical Sciences Shiraz Iran; ^2^ Jefferson Comprehensive Epilepsy Center, Department of Neurology Thomas Jefferson University Philadelphia Pennsylvania USA

To the Editor,

Recently, we published a paper that suggested that *FKBP5* single‐nucleotide polymorphisms may play a role in functional seizures (psychogenic nonepileptic seizures/attacks).^1^ We also published another paper entitled “*FKBP5* blockade may provide a new horizon for the treatment of stress‐associated disorders: An in‐silico study” providing a list of the currently available and approved drugs (e.g., fluticasone propionate, prednisolone, dexamethasone, mirtazapine, sertraline, fluoxetine, and citalopram) that could potentially be used for this purpose.^2^

More recently, I read an article entitled: “Cannabidiol alleviates neuroinflammation and attenuates neuropathic pain via targeting FKBP5.”^3^ In this study, cannabidiol (CBD) was identified as an antagonist of *FKBP5* and *FKBP5* was an endogenous target of CBD.^3^ This is a very interesting study that opens a very intriguing avenue for future research.

FK506‐binding protein 51 (*FKBP5/FKBP51*) is a gene that encodes FKBP51 protein and contributes to the regulation of glucocorticoid receptor sensitivity. Mutant variants of *FKBP5* have been associated with reduced glucocorticoid receptor sensitivity, which can lead to decreased hypothalamus–pituitary–adrenal (HPA) axis negative feedback and subsequent maintenance of glucocorticoids in the absence of threats and stress.^4^ Accordingly, *FKBP5* polymorphisms have been associated with various stress‐associated disorders such as major depression and post‐traumatic stress disorder (PTSD), and potentially with functional seizures (psychogenic nonepileptic seizures/attacks).^1^, ^5^ Therefore, *FKBP5* blockade may hold promise as a treatment option for stress‐associated disorders, including potentially functional seizures (psychogenic nonepileptic seizures/attacks).^2^

At the moment, psychotherapy is considered the best treatment option for patients with functional seizures (psychogenic nonepileptic seizures/attacks). But a large clinical trial failed to demonstrate that cognitive behavioral therapy (CBT) has a statistically significant advantage compared with standardized medical care alone for the reduction of monthly seizures in these patients.^6^ Functional seizures (psychogenic nonepileptic seizures/attacks) have devastating effects on patients' lives^7^ and therefore, the scientific community should try to discover efficacious therapeutic options (e.g., a drug) for the treatment of this condition.

In conclusion, the suggestion that CBD is an antagonist of *FKBP5* and *FKBP5* is an endogenous target of CBD could open a new horizon for future research in discovering a treatment option for patients with functional seizures (psychogenic nonepileptic seizures/attacks).

## AUTHOR CONTRIBUTIONS

The sole author had responsibility for all parts of the manuscript.

## FUNDING INFORMATION

This research did not receive any specific grant from funding agencies in the public, commercial, or not‐for‐profit sectors.

## CONFLICT OF INTEREST STATEMENT

Honorarium: Cobel Daruo, Actoverco; Royalty: Oxford University Press (Book publication).
